# Role of Endocytosis in Localization and Maintenance of the Spatial Markers for Bud-Site Selection in Yeast

**DOI:** 10.1371/journal.pone.0072123

**Published:** 2013-09-05

**Authors:** Shanshan Tuo, Kenichi Nakashima, John R. Pringle

**Affiliations:** Department of Genetics, Stanford University School of Medicine, Stanford, California, United States of America; Institute of Biology Valrose, France

## Abstract

The yeast *Saccharomyces cerevisiae* normally selects bud sites (and hence axes of cell polarization) in one of two distinct patterns, the axial pattern of haploid cells and the bipolar pattern of diploid cells. These patterns depend on distinct sets of cortical-marker proteins that transmit positional information through a common signaling pathway based on a Ras-type GTPase. It has been reported previously that various proteins of the endocytic pathway may be involved in determining the bipolar pattern but not the axial pattern. To explore this question systematically, we constructed and analyzed congenic haploid and diploid deletion mutants for 14 genes encoding proteins that are involved in endocytosis. The mutants displayed a wide range of severities in their overall endocytosis defects, as judged by their growth rates and abilities to take up the lipophilic dye FM 4–64. Consistent with the previous reports, none of the mutants displayed a significant defect in axial budding, but they displayed defects in bipolar budding that were roughly correlated with the severities of their overall endocytosis defects. Both the details of the mutant budding patterns and direct examination of GFP-tagged marker proteins suggested that both initial formation and maintenance of the normally persistent bipolar marks depend on endocytosis, as well as polarized exocytosis, in actively growing cells. Interestingly, maintenance of the bipolar marks in non-growing cells did not appear to require normal levels of endocytosis. In some cases, there was a striking lack of correlation between the overall severities of the general-endocytosis defect and the bud-site selection defect, suggesting that various endocytosis proteins may differ in their importance for the uptake of various plasma-membrane targets.

## Introduction

Cell polarization is an important characteristic of most cell types and involves both the selection of a polarization axis and the subsequent asymmetric organization of the cytoskeleton, intracellular organelles, and plasma-membrane components along this axis [1]. The yeast *Saccharomyces cerevisiae* provides an outstanding model for studies of the mechanisms of cell polarization [2], [3]. Axis selection is nonrandom and equates to bud-site selection, which in wild-type cells occurs in either of two distinct patterns, axial and bipolar, depending on the cell mating type [2], [4], [5]. During exponential growth, normal haploid cells (Mat**a** or Matα) use the axial pattern, in which each cell's first bud is made adjacent to the birth scar that marks its site of separation from its mother, and each subsequent bud is made adjacent to the immediately preceding division site. In contrast, normal diploid cells (Mat**a**/α) use the bipolar pattern, in which the first bud is almost always formed at the pole distal to the birth scar, whereas subsequent buds can be formed at either pole. These two patterns depend on a common signaling pathway based on the Ras-like GTPae Rsr1/Bud1 [2], [3], [6], [7] but on distinct cortical cues provided by different sets of marker proteins. The axial pattern depends on a transient marker provided by Bud3, Bud4, Axl2/Bud10, and Axl1 [2], [5], [8]–[16], whereas the bipolar pattern depends on persistent markers provided by Bud8, Rax1, and Rax2 at the birth-scar-distal pole and Bud9, Rax1, and Rax2 at the birth-scar pole [2], [5], [17]–[26]. Among these marker proteins, only Axl1 is haploid-specific in its expression [9], [14], [27].

It has also been reported that mutants defective in several actin-patch-associated proteins have defects in bipolar budding [28]–[33]. These proteins are now known to be involved in endocytosis, the complex process by which cells internalize extracellular fluids, particles, and plasma-membrane components [34]–[39]. Endocytosis can be divided into several stages according to the dynamics of protein composition and membrane movement at the endocytic site. In the first stage, the plasma membrane has very limited motility, and endocytic proteins and adaptors (including clathrin, Sla1, Sla2, and End3 in yeast) appear in a patch, followed by Arp2/3 complex regulators, which serve to nucleate actin polymerization. These proteins initially remain relatively immotile near the cell cortex. Near the end of this stage, actin-binding proteins (including Arp2/3, Abp1, Sac6/fimbrin, and capping proteins) appear at the site, representing the first step in actin assembly. In the next stage, the plasma membrane invaginates under the forces produced by actin polymerization and the actin-associated proteins. The final stage is highly dynamic, as membrane scission occurs to create an endocytic vesicle, endocytic proteins begin to leave the vesicle, and the vesicle fuses with endosomes.

The apparent involvement of endocytosis proteins in bipolar budding suggested that the observed persistence of the bipolar markers might not be a static state but rather one involving a dynamic interplay between polarized exocytosis and endocytosis. To explore this possibility, we constructed and analyzed a set of haploid and homozygous-diploid deletion mutants for 14 endocytosis-related genes. The results help to clarify the role of endocytosis in the generation and maintenance of the bipolar-budding markers and also suggest that various endocytosis proteins may differ in their importance for the uptake of various plasma-membrane targets.

## Materials and Methods

### Strains, plasmids, growth conditions, and genetic methods

Strain KNY408 was constructed by several crosses starting with *bud9Δ::HIS3* strain YHH614 [20] and strains in which *BUD8* or *AXL2* had been deleted as described below for the endocytosis genes. The other mutant yeast strains used in this study (Table 1) were constructed using the PCR method [40] in strain YEF473 [41] with plasmid pFA6a-His3MX6 or pFA6a-TRP1 [42] as template. Proper integration of the transformation cassette was confirmed by colony PCR of genomic DNA. The heterozygous diploids were sporulated, and Mat**a** and Matα haploid deletion strains were mated to obtain the homozygous diploids. Plasmids used in this study are described in Table 2. Standard yeast-genetic and molecular-biology methods were used [43], [44]. *Escherichia coli* strain DH12S (Life Technologies) was used as a host for plasmids. Except where noted, yeast cells were grown at ∼24°C in synthetic complete (SC) medium with 2% glucose as carbon source; specific nutrients were omitted as needed to maintain plasmids [43]. Some experiments used YM-P rich, buffered liquid medium [45] with 2% glucose as carbon source except where noted.

**Table 1:** *S. cerevisiae* strains used in this study a.

Strain	Genotype	Source
YEF473A	**a** *his3-Δ200 leu2-Δ1 lys2-801 trp1-Δ63 ura3-52* **a** */α his3-Δ200/his3-Δ200 leu2-Δ1/leu2-Δ1 lys2–801/lys2–*	37
YEF473	801 trp1-Δ63/trp1-Δ63 ura3–52/ura3–52 *bud8Δ::TRP1/bud8Δ::TRP1 bud9Δ::HIS3/bud9Δ::HIS3*	37
KNY408	axl2Δ::His3MX6/axl2Δ::His3MX6	See text
STY852	**a** *abp1Δ::His3MX6*	See text
STY842	**a** *ede1Δ::His3MX6*	See text
STY891	**a** *myo5Δ::His3MX6*	See text
STY129	**a** *cap1Δ::His3MX6*	See text
STY836	**a** *sla1Δ::His3MX6*	See text
STY92	**a** *swa2Δ::His3MX6*	See text
STY838	**a** *sla2Δ::His3MX6*	See text
STY824	**a** *chc1Δ::His3MX6*	See text
STY846	**a** *las17Δ::His3MX6*	See text
STY822	**a** *end3Δ::His3MX6*	See text
STY854	**a** *sac6Δ::His3MX6*	See text
STY850	**a** *rvs167Δ::TRP1*	See text
STY848	**a** *rvs161Δ::His3MX6*	See text
STY867	**a** *vrp1Δ::His3MX6*	See text
STY414	**a** */α abp1Δ::His3MX6/abp1Δ::His3MX6*	See text
STY419	**a** */α ede1Δ::His3MX6/ede1Δ:: His3MX6*	See text
STY909	**a** */α myo5Δ::His3MX6/myo5Δ::His3MX6*	See text
STY1177	**a** */α cap1Δ::His3MX6/cap1Δ::His3MX6*	See text
STY61	**a** */α sla1Δ::His3MX6/sla1Δ::His3MX6*	See text
STY36	**a** */α swa2Δ::His3MX6/swa2Δ::His3MX6*	See text
STY1179	**a** */α sla2Δ::His3MX6/sla2Δ::His3MX6*	See text
STY57	**a** */α chc1Δ::His3MX6/chc1Δ::His3MX6*	See text
STY58	**a** */α las17Δ::His3MX6/las17Δ::His3MX6*	See text
STY56	**a** */α end3Δ::His3MX6/end3Δ::His3MX6*	See text
STY60	**a** */α sac6Δ::His3MX6/sac6Δ::His3MX6*	See text
STY46	**a** */α rvs167Δ::TRP1/rvs167Δ::TRP1*	See text
STY59	**a** */α rvs161Δ::His3MX6/rvs161Δ::His3MX6*	See text
STY869	**a** */α vrp1Δ::His3MX6/vrp1Δ::His3MX6*	See text

a All strains are congenic to YEF473A or YEF473 (see Materials and Methods). The STY strains are arranged in order of their growth rates (as in the figures).

**Table 2:** Plasmids used in this study.

Plasmid	Description	Source
YCpHA-BUD8–2	Low copy, *LEU2, BUD8* with triple HA tag	This study a
YCp-BUD9	Low copy, *LEU2, BUD9*	[20]
YCp-2xGFP-BUD8	Low copy, *URA3*, *BUD8* with double *GFP* tag	This study b
RAX2-GFP	Low copy, *URA3, RAX2-GFP*	Gift from A. Fujita

a Like YCpHA-BUD8 [20] except in a YEplac111 [53] background.

b Like YEpGFP*-BUD8 [20] except with two in-frame copies of the GFP coding sequence and in a YCplac33 [53] background.

### Growth-rate measurements

To determine doubling times in liquid medium, cells were grown to exponential phase (OD_600_ = 0.3–0.6). The OD_600_ was recorded, the culture was diluted 2-fold with fresh medium, and the time required to grow back to the original OD_600_ was determined.

### Staining and microscopy

To visualize birth and bud scars, cells were stained with 0.2 mg/ml Calcofluor without fixation [17] and observed with a Nikon Eclipse 600 FN microscope equipped with a Hamamatsu ORCA-2 CCD camera and an Apo 100×/1.40 NA oil-immersion objective. Cells expressing GFP-tagged proteins or stained with FM 4–64 were observed using the same microscope. All images were collected using Metamorph software (Molecular Devices). Exposure times were ∼30 ms (Calcofluor), ∼2.5 s (GFP), and ∼100 ms (FM4-64).

### Evaluation of endocytosis defects by FM 4–64 uptake

Cells were grown in YM-P medium to exponential phase and collected by centrifugation at ∼1,200 *g* for 1 min. The pellets were resuspended in 100 µl of 5 µM FM 4–64 in water and incubated on ice for 10–12 min. Samples were examined by fluorescence microscopy (time 0), and the remaining cells were then collected by centrifugation (as above), released in 4 ml of fresh YM-P medium at ∼24°C, and examined by fluorescence microscopy after 5 min. Only one or two strains were included in each experiment to ensure that all samples were treated similarly.

### Scoring budding patterns

To assess the overall budding patterns of haploid cells, cells with three bud scars were scored for whether those bud scars were or were not all clustered at the birth-scar-proximal pole. To assess the overall budding patterns of diploid cells, cells with ≥5 bud scars were classified into three groups: bipolar (all bud scars at the poles, at least one scar at each pole); random (≥1 bud scar in the equatorial region); and unipolar (all bud scars clustered at only one pole). To score the positions of first, second, and third buds on diploid cells, cells with a growing bud and the appropriate numbers of bud scars (no bud scar for first-bud position; one bud scar for second-bud position; etc.) were scored for the position of the growing bud relative to the birth scar.

## Results

### Relative severities of endocytosis defects in endocytosis mutants

**Figure 1:**
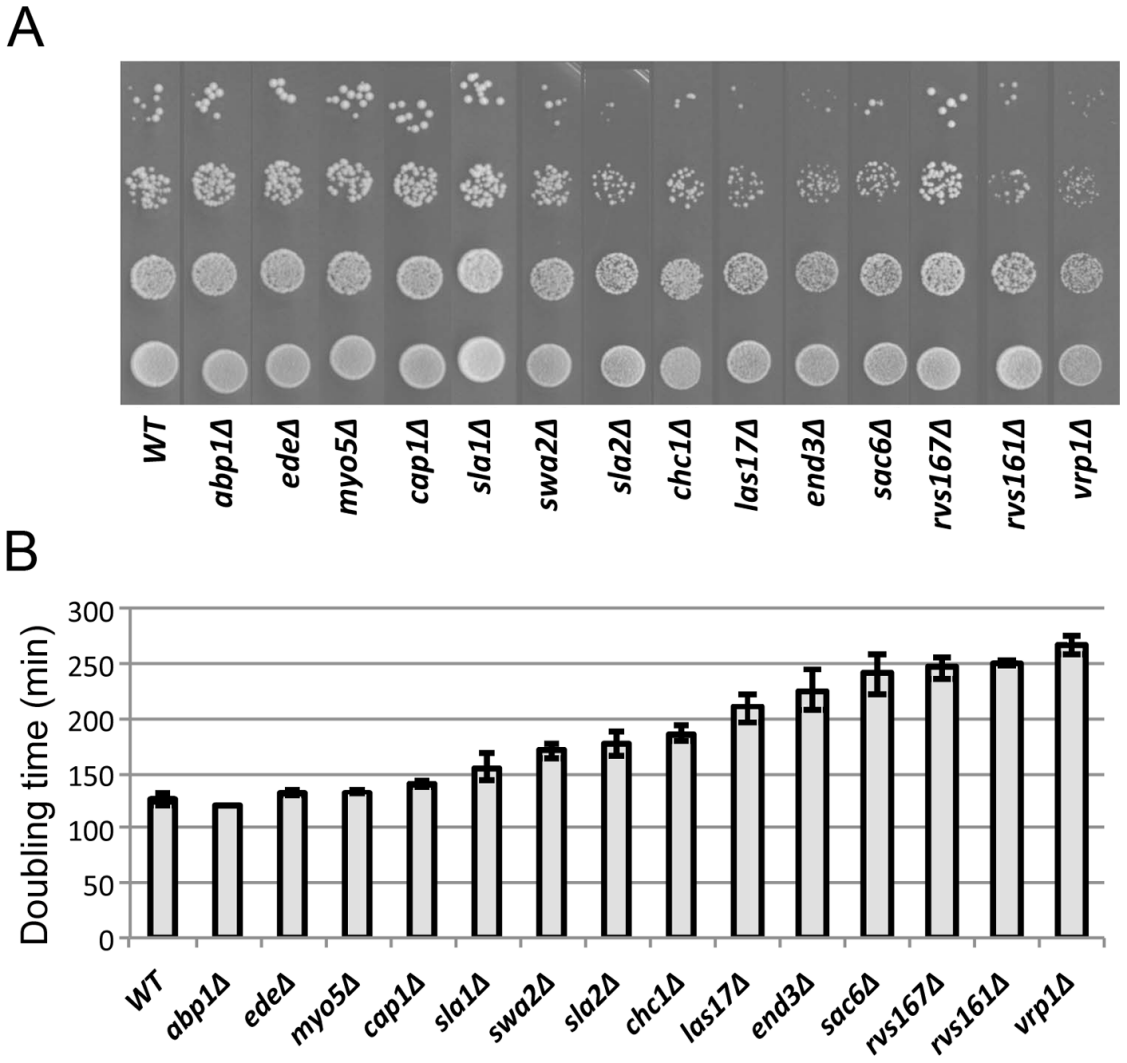
Growth rates of wild-type and mutant diploid strains. (A) Strains (Table 1) were grown overnight to stationary phase in SC medium, spotted in a 10-fold dilution series onto SC plates, and incubated at 24°C for 3 days. (B) Doubling times in SC liquid medium at 24°C were determined as described in Materials and Methods. Means and standard deviations from three independent experiments are shown for each strain.

To investigate the role of endocytosis in bud-site selection, we generated a set of haploid and homozygous-diploid deletion mutants for 14 endocytosis-related genes in a common strain background (Table 1). We first evaluated the relative severities of the endocytosis defects in these mutants both by determining their growth rates on solid and liquid medium and by monitoring uptake of the lipophilic dye FM 4–64. The mutants displayed a considerable range of growth rates, from essentially the same as wild type to much slower, and the colony growth rates observed on solid medium were generally well correlated with the doubling times determined in liquid medium (Figure 1A, B). (In the one clear exception, the *rvs167Δ* strain appeared to grow significantly better on solid medium, for unknown reasons.) We used the relative growth rates in liquid medium to organize the data presentation in Figure 1 and the subsequent figures.

**Figure 2:**
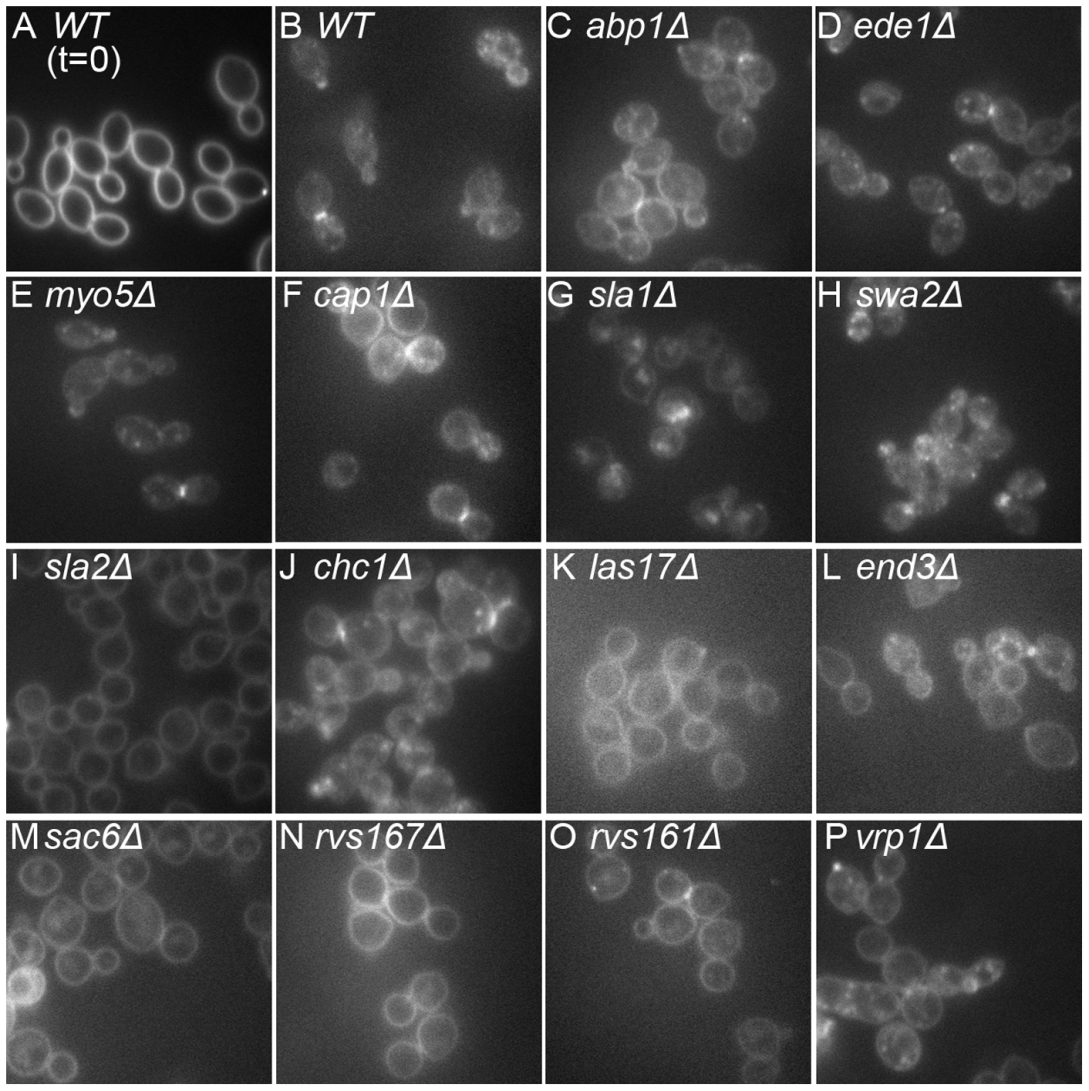
Fluid-phase endocytosis defects of mutant diploid strains. Uptake of the lipophilic dye FM 4–64 was examined as described in Materials and Methods. Cells were photographed after labeling on ice (A) or 5 min after release into fresh medium at 24°C (B–P). Strains are shown in the order of their growth rates (Figure 1B).

We then examined fluid-phase endocytosis by monitoring the uptake FM 4–64 (see Materials and Methods). Mutant and wild-type cells that had been labeled on ice to inhibit endocytosis showed a uniform plasma-membrane labeling (Figure 2A and data not shown). After release into fresh medium at 24°C for 5 min, wild-type cells had taken up most of the dye into internal vesicles (Figure 2B). The mutants showed varying degrees of internalization defects that could be roughly categorized as follows: little or no defect (FM 4–64 fluorescence mostly in internal vesicles), *ede1Δ* and *myo5Δ* (Figure 2D, E); moderate defect (FM 4–64 fluorescence on both the plasma membrane and internal vesicles), *abp1Δ*, *cap1Δ*, *sla1Δ*, *swa2Δ*, *chc1Δ* and *vrp1Δ* (Figure 2C, F, G, H, J, P); and severe defect (FM 4–64 fluorescence mostly still on the plasma membrane), *sla2Δ*, *las17Δ*, *end3Δ*, *sac6Δ*, *rvs167Δ*, and *rvs161Δ* (Figure 2I, K, L, M, N, O). As expected, the severities of the growth-rate and FM 4–64-uptake deficiencies were approximately correlated.

### Defective bipolar-bud-site selection in endocytosis mutants

**Figure 3:**
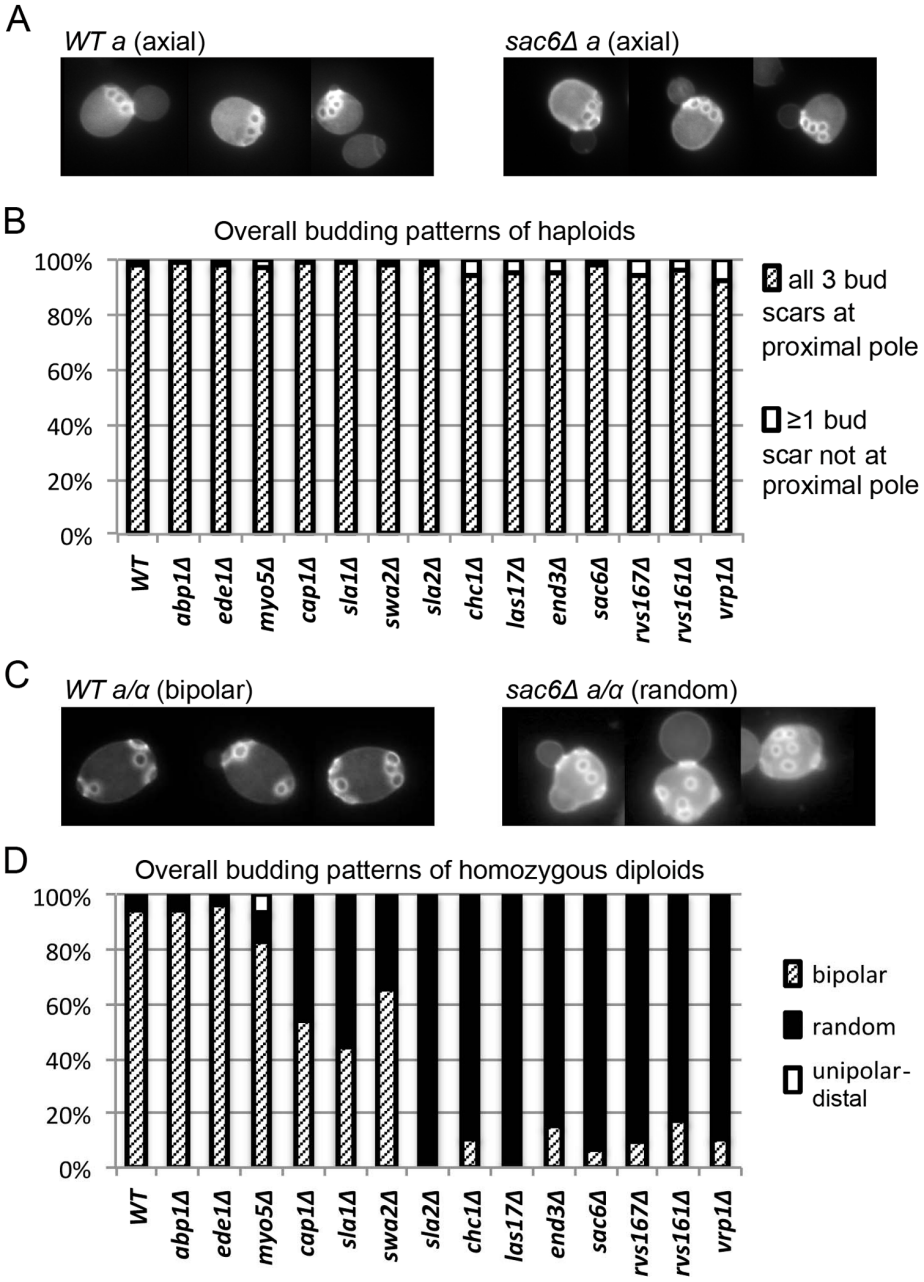
Effects of endocytosis defects on the overall budding patterns of haploid and diploid strains. Cells of wild-type and mutant strains (Table 1) growing exponentially in SC medium were stained with Calcofluor to visualize bud scars. (A) Normal axial budding of wild-type haploid cells (left) and the haploid *sac6Δ* mutant (right). (B) Quantification of the overall budding patterns of haploid cells with three bud scars (see Materials and Methods). 100 cells were scored for each strain. (C) Normal bipolar budding of wild-type cells (left) and random budding of the diploid *sac6Δ* mutant (right). (D) Quantification of overall budding patterns of cells with ≥5 bud scars (see Materials and Methods). At least 100 cells were scored for each strain. No significant numbers of cells with unipolar-proximal patterns were seen with any strain.

To investigate the possible roles of endocytosis in the generation and/or maintenance of the spatial markers that guide bud-site selection, we examined the effects of the 14 deletion mutations on the budding patterns of both haploid and diploid cells. All of the haploid mutants showed seemingly normal axial budding (Figure 3A, B), as reported previously for *abp1, cap1, ede1, end3, las17, rvs161, rvs167, sac6, sla1, sla2, swa2*, and *vrp1* mutants [28]–[33], [46], [47]. In contrast, when we examined the homozygous diploids, we observed defects in the overall budding patterns of older cells (≥5 bud scars) that were moderately well correlated with the severities of the endocytosis defects as judged by growth rate and FM 4–64 uptake (Figure 3C,D). However, the transitions in the severities of the budding-pattern defects appeared more abrupt than might have been expected from the small differentials in endocytosis defects (Figures 1 and 2), suggesting that the budding-pattern defects are not simply secondary effects of the reduced growth rates.

**Figure 4:**
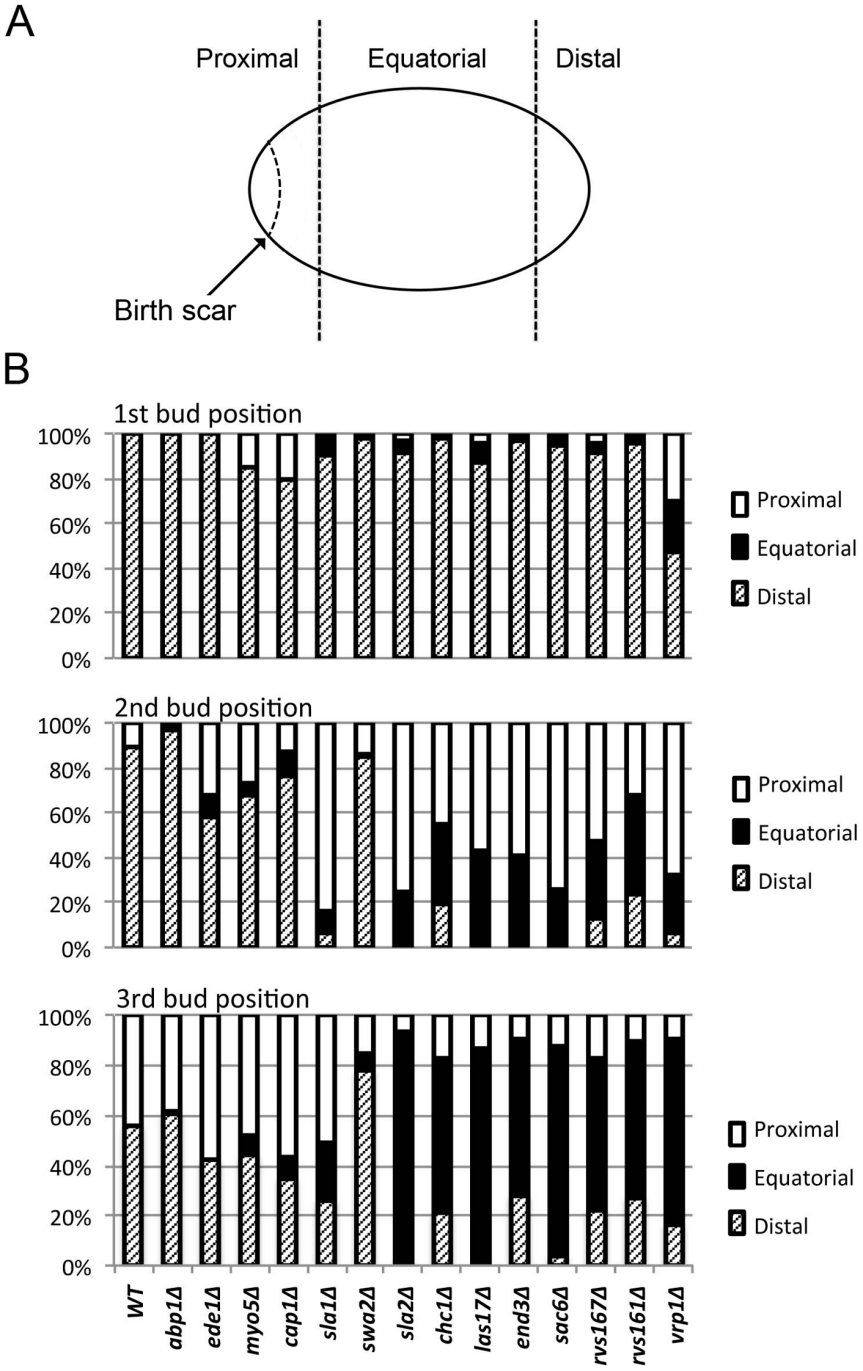
Effects of endocytosis defects on bud position in diploid cells. (A) Schematic representation of the cell cortex showing birth-scar-proximal, equatorial, and birth-scar-distal regions as defined for scoring. The distances from the boundaries of the equatorial region to the birth scar and the distal tip are in each case approximately the diameter of one bud scar. A bud scar that was either wholly or partly within a polar region was scored as being at that pole. (B) Quantification of bud positions (see Materials and Methods) in wild-type and mutant diploid strains (Table 1). At least 100 cells were scored for each count.

To obtain a more detailed picture of the effects of endocytosis defects on the bipolar budding pattern, we examined the positions of first, second, and third buds relative to the birth scar (Figure 4A). Remarkably, with the exception of *vrp1Δ* (whose severe morphological defects make its budding patterns hard to interpret), all of the mutants showed little difference from wild type in their ability to position the daughter cell's first bud at the birth-scar-distal pole (Figure 4B, top panel). This might mean that the endocytosis machinery is not involved in the initial generation of the bipolar marker at the distal pole of the newborn daughter cell. Alternatively, it might mean that a weaker-than-normal marker is established at the distal pole when the endocytosis machinery is defective, but that even such a weakened marker is sufficient to direct most budding events to that pole if no other strong signal is present. It is known that the axial-budding marker cannot be used effectively in diploid cells because of the absence of Axl1 [9], [14], [27], and there is some evidence that the bipolar marker at the proximal pole (involving Bud9, Rax1, and Rax2; see Introduction) requires some kind of maturation before it is effective [5], [20]. Further evidence in support of this hypothesis was provided by an experiment in which cells expressing only Bud8 or only Bud9 were examined for bud position before and after a period of starvation. Strikingly, although the Bud8-containing distal-pole marker was fully competent to direct budding events in newborn daughter cells as well as in daughter cells that had been starved for varying periods (Table 3), the Bud9-containing proximal-pole marker was able to direct a larger fraction of budding events in daughter cells that had been starved for 2–20 d than it was in newborn daughter cells (Table 3).

**Table 3:** Persistence of bipolar-budding signals during starvation and the importance of a maturation step for the Bud9-containing signal a.

	First-bud position (% distal pole: % equatorial: % proximal pole)		
Days of starvation	No plasmid	YCpHA-BUD8–2	YCp-BUD9
0	24∶70∶6	93∶6∶1	11∶22∶67
2	N. D.	95∶5∶0	3∶7∶90
10	21∶72∶7 b	92∶8∶0	2∶3∶95
20	24∶73∶3	86∶13∶1	2∶4∶94

a A strain lacking all bud-site-selection signals (KNY408; Table 1) was transformed with the indicated plasmids to restore the distal-pole or proximal-pole bipolar signal, grown to exponential phase in SC-Leu medium at 24°C, and scored for the positions of first buds on daughter cells (day 0). Cells were then transferred into YM-P medium (0.2% glucose) at ∼10^8^ cells/ml and incubated at 24°C with continuous shaking for several weeks. At intervals, samples of cells were washed, re-suspended in fresh SC-Leu medium, incubated at 24°C until numerous new buds began to appear, and scored for the positions of the first buds on daughter cells.

b Cells were released and scored after 8 rather than 10 days of starvation.

**Figure 5:**
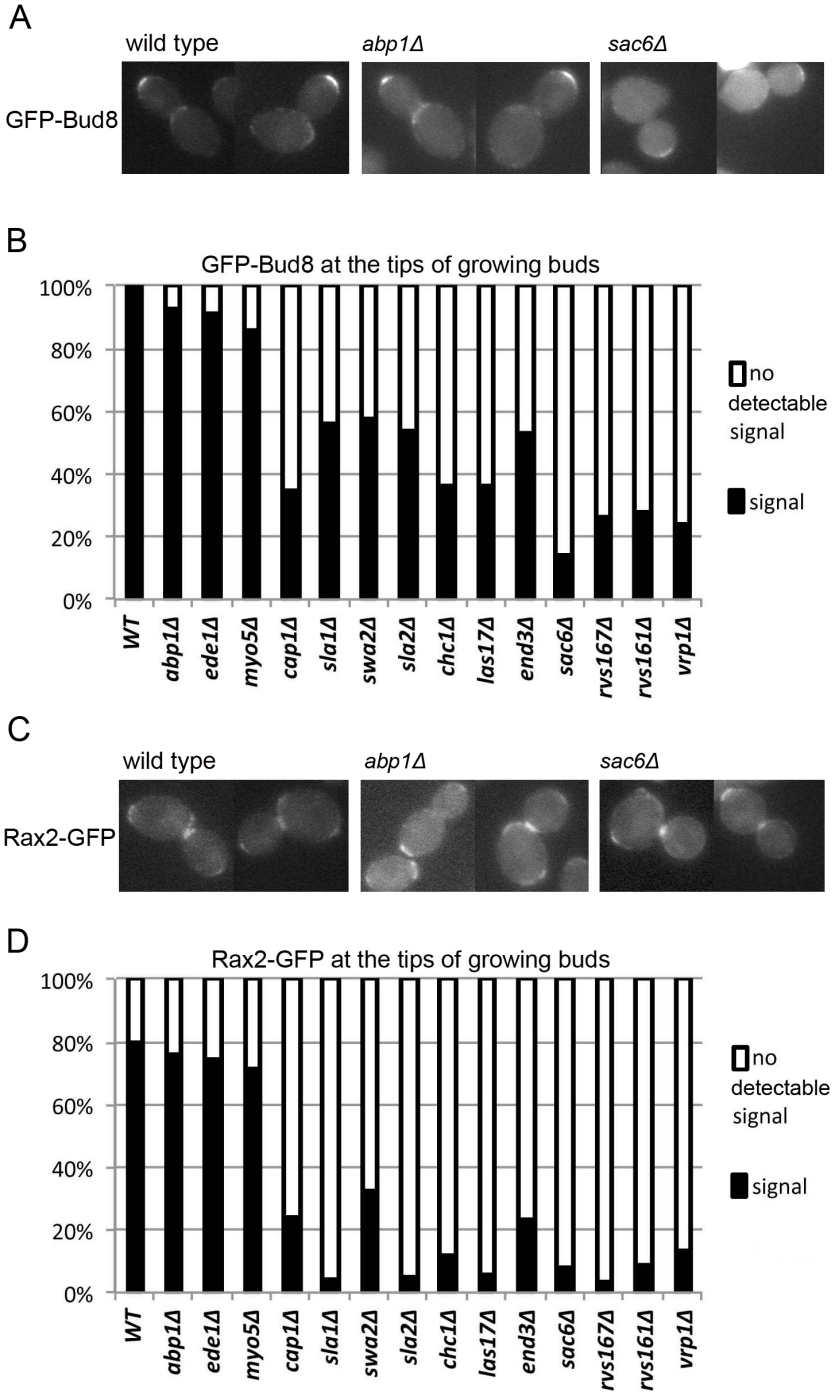
Effects of endocytosis defects on the localization of Bud8 and Rax2. Wild-type and mutant diploid strains (Table 1) were transformed with low-copy plasmids (Table 2) expressing GFP-Bud8 (A and B) or Rax2-GFP (C and D), grown to exponential phase in SC-Ura medium at 24°C, and examined for the presence or absence of detectable GFP signal at bud tips. (A) Cells of the indicated strains expressing GFP-Bud8. These cells provide examples of cells scored as positive for GFP-Bud8 signal (even though such cells were in the minority in the *sac6Δ* strain). (B) ≥100 cells with a growing bud were scored for each GFP-Bud8-expressing strain. (C) Cells of the indicated strains expressing Rax2-GFP. These cells provide examples (i) of cells scored as positive for Rax2-GFP signal (wild type and *abp1Δ* cells) and (ii) of cells scored as negative (*sac6Δ* cells). (D) 60–100 cells with a growing bud were scored for each Rax2-GFP-expressing strain. In both B and D, every budded cell observed was scored.

To directly evaluate the strength of the distal-pole marker in the endocytosis mutants, we examined the localization of its components Bud8 and Rax2. For ease of quantification, we scored the presence or absence of detectable patches of the GFP-tagged proteins at the tips of growing buds, which will become the distal poles of the newborn daughter cells; the rather weak fluorescence signals are more consistently detected at the bud tips than on the daughter cells themselves. An inability to detect a signal might mean either that less total protein is targeted to the cell tip in a given strain and/or that the cell-surface patches of protein are more diffuse and thus more difficult to detect. In the three mutants with the weakest endocytosis defects, we could detect both the GFP-Bud8 and Rax2-GFP signals with efficiencies similar to those in wild-type cells (Figure 5). For the other 11 mutants, signal detection was substantially reduced, particularly for Rax2-GFP, and there was only a rough correlation between the extent of signal reduction and the severity of the endocytosis defect as judged by other measures (Figures 1 and 2). These results suggest that active endocytosis, as well as polarized delivery, is necessary to form a normal patch of the distal-pole marker and supports the hypothesis that even a patch that is substantially less robust than normal can direct most polarization-initiation events in a newborn diploid daughter cell where other potential markers are weak.

We next asked about the maintenance of the distal-pole marker into the second and third budding cycles. In wild-type diploid cells of this strain background, ∼90% of second buds and more than half of third buds are also formed at the distal pole [5], [17], [20] (Figure 4B, middle and bottom panels). In contrast, most of the mutants were at least partially defective in positioning their second and third buds at the distal pole (Figure 4B, middle and bottom panels). These data suggest that a dynamic process involving endocytosis as well as polarized exocytosis is involved in maintaining the bipolar-budding marker at the distal pole. The severities of the defects in marker maintenance were approximately correlated with those of the endocytosis defects as judged by other measures (Figures 1 and 2), except for the *cap1, sla1, swa2*, and *sla2* mutants, in which small differences in endocytosis defects were accompanied by large differences in budding-pattern defects. Interestingly, the severe budding-pattern defects of the *sla1* and *sla2* mutants paralleled their strong effects on the cells' ability to form a well defined patch of Rax2 (Figure 5D). Taken together, the results suggest that the components of the canonical endocytosis pathway are differentially involved in the formation and/or maintenance of the patches of the distal-pole bipolar-budding marker.

Also interesting were the mutants' abilities to use the proximal pole for budding. In wild-type cells, nearly all second and third buds that are not at the distal pole are at the proximal pole (Figure 4B, middle and bottom panels). This was also true of the five mutants (*abp1Δ – sla1Δ*) with the least severe endocytosis defects. Even the mutants with more severe endocytosis defects showed effective use of the proximal pole in the second cell cycle (Figure 4B, middle panel), although by the third cycle bud positions were essentially random (Figure 4B, bottom panel). Thus, as with the distal-pole marker, it appears that the mutant cells have some success in originally depositing the proximal-pole marker but then cannot maintain it. An interesting special case was the *swa2Δ* mutant, whose persistent budding at the distal pole even into the third cell cycle (Figure 4B, middle and bottom panels) suggests that it is either less effective in establishing the proximal-pole marker or less defective in maintaining the distal-pole marker, compared to other mutants with similar overall defects in endocytic ability.

**Figure 6:**
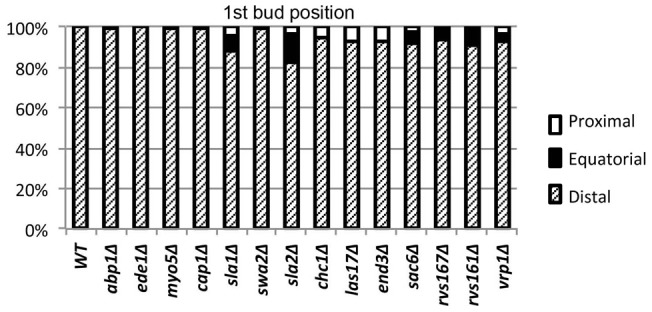
Positions of first buds on wild-type and mutant diploid cells after a period of starvation. Wild-type and mutant strains (Table 1) were inoculated into YM-P medium containing only 0.2% glucose at ∼10^8^ cells/ml and incubated for 5 days. Cells were then sonicated briefly, collected by brief centrifugation, resuspended in YM-P medium containing 2% glucose, and scored after 3–5 h for the positions of emerging buds on daughter cells (*i.e.,* cells with no previous bud scars). ≥100 cells were scored for each strain, and all daughter cells with emerging buds were scored.

One of the original indications that potential bipolar-budding sites are marked by stable markers was the observation that daughter cells budded normally at their distal poles after a period of starvation followed by re-feeding [5] (Table 3). We performed a similar experiment to ask if maintenance of the distal-pole signal during starvation were a dynamic process involving endocytosis. Remarkably, during 5 d of starvation, the mutants did essentially as well as wild-type cells in maintaining a distal-pole marker that could effectively direct cell polarization when the cells were re-fed (Figure 6).

## Discussion

Previous studies have suggested that endocytosis-related genes are important for the normal bipolar budding of diploid cells but not for the axial budding of haploid cells [28]–[33], [46], [47]. However, the function(s) of endocytosis in bud-site selection had not been investigated in detail. To explore this matter further, we constructed and analyzed congenic mutants of 14 endocytosis-related genes, including the 12 included in the earlier studies plus two others. Consistent with the earlier studies, we found that none of the mutations significantly affected axial budding but that most of the mutations (except for a few with seemingly weak effects on endocytosis) had pronounced effects on bipolar budding. Our results suggest several conclusions.

First, because the Rsr1/Bud2/Bud5 pathway that connects the axial and bipolar spatial markers at the cell surface to Cdc42 and other components of the cell-polarization machinery appears to be identical in haploid and diploid cells [2], [7], [48], [49], it appears (not surprisingly) that the role of endocytosis in bipolar bud-site selection must be exerted at the level of the cell-surface markers involving the plasma-membrane proteins Bud8, Bud9, Rax1, and Rax2 [2], [17]–[26].

Second, although the axial-budding marker also involves a plasma-membrane glycoprotein, Axl2 [2], [11], [12], [50], it appears that the endocytosis machinery is not involved in the establishment or maintenance of the patch of axial marker at the mother-bud neck. The short lifetime of the axial marker [2], [5], [10], [12], [13], [15], [16], [50] may make a process of dynamic maintenance unnecessary, and the endocytic machinery may instead be involved in the removal of the axial signal early in the new cell cycle.

Third, observations of GFP-Bud8 and Rax2-GFP appear to demonstrate that endocytosis proteins are involved in the initial establishment of a normal patch of bipolar-budding marker, implying that formation of this patch is a dynamic process that involves effective endocytosis as well as polarized exocytosis [2], [20], [21], [51]. This process seems likely to involve the “corralling” by endocytic activity of the patch of bipolar-marker proteins, as recently described by Jose *et al*. [52] for proteins such as Cdc42 that lie downstream of the bud-site-selection proteins in the yeast polarity-establishment pathway. Moreover, the observation that the endocytosis mutants position nearly all first buds normally at the distal pole, despite the seemingly reduced concentration of marker there, suggests that in wild-type daughter cells, the signal from the distal-pole marker is substantially stronger than it needs to be to direct polarization events to that pole. This conclusion is consistent with other evidence that the bipolar marker at the proximal pole is weak in newborn daughter cells and requires maturation by a process whose details remain unknown (see Results).

Fourth, the dramatically decreased ability of the mutants with strong endocytosis defects to use the distal pole in their second and third budding cycles suggests that maintenance of the distal-pole marker during active growth requires a dynamic process involving endocytosis. Alternatively, it may be that an initially weak signal established at the distal pole when endocytosis is defective is sufficient to recruit polarization events to that site when no other strong signal is present, but that as the Bud9-dependent proximal-pole marker matures during the first cell cycle, it can compete effectively for the recruitment of the polarization machinery. Indeed, it seems likely that both of these factors are in play. In the second budding cycle, even the more severely affected endocytosis mutants position many buds at the proximal pole, suggesting that the marker at this pole has become strong relative to that at the distal pole. However, by the third budding cycle, most buds are positioned randomly in these same mutants, suggesting that by this point, both the Bud8-dependent distal-pole marker and the Bud9-dependent proximal-pole marker have decayed. Moreover, the observation that starved mutant cells to show a very strong preference for the distal pole upon resuming growth suggests both that dynamic remodeling is not necessary to maintain the distal-pole marker in non-growing cells and that maturation of the proximal-pole marker is not sufficient for it to predominate in the recruitment of the polarization machinery unless the distal-pole marker has also decayed.

Finally, the absence of a simple correlation between the overall severity of the endocytosis defect (as measured by growth rate and the efficiency of FM 4–64 uptake) and the severity of the bud-site selection defect suggests that the various proteins of the canonical endocytosis pathway [34]–[39] do not participate equally in the establishment and/or maintenance of the distal-pole marker, and thus, more broadly, that that the various endocytosis proteins may differ in their importance for the uptake of various plasma-membrane proteins or domains.
